# The St. Louis Meeting

**Published:** 1874-09

**Authors:** 


### EDITORIAL. ETC.

---

*The St. Louis Meeting.*—We regret to learn that the late meeting of the Southern Dental Association was very slimly attended, but few dentists outside of St. Louis being present, especially practitioners from the Southern States. This was quite a disappointment to the friends of the Association, for it was expected that many of our professional brethren from the West, Southwest and upper Mississippi would have been present, and rendered it one of the most interesting meetings ever held. Was it too far from home? It appears to have been quite a mistake to go so far west, and we think an equally grave mistake has been committed in selecting Memphis for the next meeting, as the place should have been farther from the scene of the recent one, after which it would have been proper to have selected Memphis at a suitable season of the year. Louisville, Nashville, or some city in the mountainous parts of North Carolina or Georgia would certainly have been more appropriate. As it is we can only hope that our fears of a meeting like that of St. Louis, so far as attendance is concerned, may prove groundless, and the Memphis meeting be a successful one. We were pleased to see the names of our friends Drs. Redman, Morgan, Arrington and Walker, among those present at St. Louis, and had it been in our power to have been with them, we no doubt would have enjoyed the reunion, as we have not forgotten the pleasant time we passed together at Atlanta, Georgia, when this Association was organized. According to a statement made by Dr. Redman, when the question of changing the name of the Association was under discussion, it appears that some object to the term "Southern." This is altogether an erroneous idea, for the Association, although it was formed more especially for Southern practitioners, has been quite liberal in its action, as the proceedings of each year will demonstrate. Northern practitioners have not only been invited to attend its meetings, but also to become members and thus participate in its work—many having done so, and been appointed members of the standing committees. Therefore this Association has not shown an illiberal spirit, or a desire to confine its work to practitioners of one section of the country, but has opened wide its doors to all respectable dentists no matter from where they came. The Association, it is true, originated in the Southern States, and was so named on account of the desire manifested to interest the practitioners of these States, few of whom ever attend any such organizations.

We sincerely hope that the members of the profession in the South will become interested in the welfare of this Association, and strive to make it what it should be.

